# Unidos na diferença: sobre a vocação da sociologia brasileira no Instituto Universitário de Pesquisas do Rio de Janeiro, 1973-1977

**DOI:** 10.1590/S0104-59702024000100008

**Published:** 2024-04-05

**Authors:** Luiz Augusto Campos, José Szwako

**Affiliations:** i Professor, Programa de Pós-graduação em Sociologia/Instituto de Estudos Sociais e Políticos/Universidade do Estado do Rio de Janeiro. Rio de Janeiro – RJ – Brasil lascampos@iesp.uerj.br; ii Professor, Programa de Pós-graduação em Sociologia/Instituto de Estudos Sociais e Políticos/Universidade do Estado do Rio de Janeiro. Rio de Janeiro – RJ – Brasil zeszwako@iesp.uerj.br

**Keywords:** History of sociology, Intellectuals, Institutionalization, Intellectual history, Instituto Universitário de Pesquisas do Rio de Janeiro (IUPERJ, História da sociologia, Intelectuais, Institucionalização, História intelectual, Instituto Universitário de Pesquisas do Rio de Janeiro (Iuperj

## Abstract

Este artigo busca compreender a vocação científica consagrada pela primeira geração (1973-1977) de pesquisadores do Programa de Pós-graduação em Sociologia do Instituto Universitário de Pesquisas do Rio de Janeiro (Iuperj). Embora o Iuperj seja visto como berço da moderna ciência política brasileira, pouco se sabe sobre sua sociologia. Para tal, baseamo-nos em documentos, entrevistas e bibliografia secundária. Queremos nuançar diagnósticos sobre essa geração, ora vista como excessivamente heterogênea, ora como pouco original se comparada à ciência política iuperjiana. Na vocação daquela geração, o elogio à especialização teórico-metodológica era parte central de uma sociologia política que buscava dar respostas às demandas de uma sociedade na encruzilhada entre modernização e redemocratização.

## Abstract (en)

This article investigates the first generation (1973-1977) of researchers trained in the Graduate Program in Sociology at the Instituto Universitário de Pesquisas do Rio de Janeiro (IUPERJ). While IUPERJ is known as the birthplace of modern Brazilian political science, sociology there is less well known. Using documentary resources, interviews, and the secondary literature, we take a nuanced look at this generation, which has been described as both excessively heterogeneous and less original in comparison to political science at IUPERJ. For them, theoretical and methodological specialization was seen as central to a political sociology that sought responses to the demands of a society at the crossroads between modernization and redemocratization.

A história das ciências sociais brasileiras tem sido contada e recontada a partir de seus supostos contrastes e das disputas por consagração. Exemplos disso são as análises que contrapõem uma antropologia extra-acadêmica e uma sociologia voltada para as faculdades e os colegiais (Corrêa, 1987; Meucci, 2000), ou os padrões de recepção e circulação internacional de ideias e autores (Villas-Bôas, 2006, 2007). A história hegemônica das ciências sociais foi contada a partir de uma lógica um tanto heroica da chamada escola sociológica paulista (Arruda, 1995; Miceli, 1995), cuja consagração acabou, direta ou indiretamente, marginalizando outras experiências nacionais e regionais. Em sentido oposto, não são poucas hoje as tentativas de recontar essa história, seja reposicionando o papel nela desempenhado pela ciência política (Leite, Codato, 2013; Lynch, 2017), seja redimensionando a visão relativamente homogeneizante da chamada “escola paulista” (Bastos, 2002), seja ainda revendo e afirmando o peso transnacional dos financiamentos e agendamentos sobre as preocupações do próprio Florestan (Cancelli, Mesquita, Chaves, 2020; Chaves, 2018; Keinert, 2011).

Nesse leque de oposições, a tensão entre Rio de Janeiro e São Paulo ganhou sua versão mais acabada na contraposição entre um elogio de inspiração francesa a Florestan e a defesa mannheimiana do Instituto Superior de Estudos Brasileiros, o Iseb (respectivamente, Miceli, 1995; Pecaut, 1990). Não raro, o Iseb foi intelectualmente rebaixado por leituras uspianas, sendo mesmo, quiçá até hoje, injustamente taxado de “fábrica de ideologias” (ver Toledo, 1977). Apesar dos *mea culpa*,^1^ coube ao Instituto Universitário de Pesquisas do Rio de Janeiro (Iuperj) a posição de algum respeito intelectual por parte dos detratores do velho Iseb: ao lado da Universidade Federal de Mina Gerais (UFMG), o Iuperj tem sido tradicionalmente reconhecido como uma das principais fontes irradiadoras da moderna ciência política no Brasil (Forjaz, 1997; Leite, Codato, 2013; Marenco, 2016; Canêdo, 2018; Amorim, 2021; Keinert, 2011).

No entanto, se a ciência política carioca conquistou tal peso, muito pouco se sabe, ainda hoje, sobre a sociologia do Iuperj e sua relevância no tipo de vocação sociológica que se consagrou no Brasil a partir dos anos 1970. Não que os pioneiros dessa disciplina no instituto sejam secundários ou marginais em nosso cânone. Ao contrário, quase todos são contemporaneamente vistos como clássicos de diversas áreas temáticas das ciências sociais brasileiras, como é o caso de Neuma Aguiar e os estudos de gênero (Caruso, 2019; Costa, 2004; Cypriano, 2013; Ramos, 2009), Simon Schwartzman e os estudos sobre a formação do nosso Estado (Bertero, 1979; Monteiro, 2009), Edmundo Campos Coelho e os estudos de diversas elites e organizações (Almeida, 2001; Freitas, 2001), Carlos Hasenbalg e as pesquisas sobre desigualdades raciais (Figueiredo, 2015; Guimarães, Hasenbalg, 2006; Lima, 2014), Nelson do Valle Silva e a área de estratificação social (Bachini, Chicarino, 2018; Barbosa et al., 2013), Luiz Antonio Machado da Silva e as pesquisas sobre movimentos urbanos e violência (Machado da Silva et al., 2018; Werneck, Teixeira, Talone, 2020), entre outros. No entanto, esses nomes raramente são estudados como um conjunto, muito menos como uma escola ou formuladores de uma dada concepção de fazer sociológico.

Assim, essa geração contrasta duplamente, seja com seus vizinhos internos da ciência política iuperjiana, ou externos, mormente da sociologia paulista. Os primeiros foram, com razão, encarados como representantes de uma ciência política neoinstitucionalista de matriz estadunidense, fortemente centrada na crítica ao ensaísmo, então prevalecente na reflexão política acadêmica (Forjaz, 1997). Apesar de suas diferentes fases e frentes, a sociologia paulista é vista como empreendimento historicamente dedicado a buscar sínteses totalizantes para explicar a modernização brasileira, quase sempre a partir de estudos de caso e referências teóricas europeias (Jackson, 2007). Já a sociologia iuperjiana seria marcada pela heterogeneidade de temáticas e uma alegada baixa originalidade epistêmica em relação à ciência política do mesmo instituto (Keinert, 2011).

Nosso objetivo aqui é nuançar esses diagnósticos por meio de dados empíricos extraídos da vida organizacional e intelectual iuperjiana. Apesar de fortemente plasmada por uma concepção de ciência dominante nos EUA, já influente dentro da ciência política do instituto, a primeira geração de sociólogos do Iuperj adaptou reflexivamente suas premissas para coaduná-las a demandas sociopolíticas específicas. O elogio à especialização temática devia servir, segundo eles, à produção incremental de um campo de conhecimento científico mais amplo, alinhado não só a problemáticas e às missões políticas de seu tempo, mas também à complexificação e à diferenciação estruturais de nossa sociedade. Aos poucos, essa geração deslocou-se de uma agenda fixada no desenvolvimento, não raro entendido como processo totalizante e por vezes teleológico, para pesquisas sobre os obstáculos à construção de uma sociedade democrática no país. Especialização àquela altura não implicava, contudo, descompromisso com os desafios colocados à ciência social pela política nacional, dilema que foi objeto explícito de reflexão dessa geração.

Diga-se de passagem, a recusa das sínteses totais parece ter sido a marca dessa geração que a impediu de ser encarada como grupo ou escola. Como veremos, se há unidade entre eles, ela não residia em uma agenda teórica compartilhada, muito menos em origens sociais comuns. Os sociólogos do Iuperj tendiam a compartilhar, antes, um projeto de vocação sociológica, centrado no elogio à especialização como resposta intelectual adequada às demandas de uma sociedade situada na encruzilhada entre modernização e democratização, quer dizer, uma sociedade que, sob um regime autoritário e por ação dele, vinha sofrendo tensas e intensas dinâmicas modernizantes observadas, sobretudo, nas grandes cidades e nas suas franjas.

Nas páginas que se seguem, servimo-nos de distintas fontes coligidas e disponibilizadas pelo projeto de celebração do cinquentenário da pós-graduação que teve seu início no Iuperj e sua continuidade, institucional e intelectual, no âmbito do Instituto de Estudos Sociais e Políticos da Universidade do Estado do Rio de Janeiro – cinquentenário completado em 2019.^2^ Além de documentos, entrevistas e bibliografia secundária, a pesquisa se baseou em três fontes primárias: os cadernos de disciplinas editados anualmente pelo instituto; anexos da revista *Dados* com relatos de pesquisas e do cotidiano do instituto; e os relatórios de gestão do Iuperj, que continham informações sobre as pesquisas desenvolvidas na instituição.

O que se segue está dividido em cinco partes. Na primeira, esclarecemos algumas das premissas teóricas que orientaram a pesquisa e o modo como se distinguem das visões dominantes presentes na literatura especializada. Na segunda, recompomos a fundação do Iuperj com ênfase na história da sociologia no instituto, presente inicialmente na formação de alguns professores e depois como programa de pós-graduação formalmente distinto. O debate sobre especialização e a preocupação com a “ação concreta” assumiu lugar central na época, o que é discutido na terceira seção. A quarta versa sobre a tradução organizacional da encruzilhada entre modernização e democratização diante de um tipo de formação intelectual que pretendia articular métodos e teoria, isto é, articular métodos específicos de pesquisa com teoria sociológica mais amplamente concebida. A quinta parte, por seu turno, discute brevemente os laços políticos desses nomes, e a sexta contém uma breve conclusão sobre esse percurso.

## A sociologia do Iuperj na sociologia das ciências sociais

Embora tenha servido como uma das matrizes para o processo de modernização da pós-graduação em ciências sociais, a sociologia do Iuperj escapa à bibliografia especializada. Uma das razões que explica isso é a premissa segundo a qual as suas dinâmicas não eram próprias, mas subsumidas à ciência política, esta sim amplamente estudada e reconhecida (Keinert, 2011). Outra explicação está no peso dado à sociologia paulista desenvolvida a partir dos anos 1940. Embora suas atividades de pesquisa e formação tenham sido brutalmente interrompidas logo após o golpe de 1964, e reestruturadas apenas muitos anos depois, ela ainda é enquadrada como matriz fundamental de nossa sociologia (Miceli, 1995, 2001 [publicado originalmente em 1989]; Jackson, 2007).

É verdade que os diagnósticos da sociologia sobre o autoritarismo e as alternativas a ele convergiam para aqueles de seus colegas politólogos. No entanto, enquanto aquela ciência política pioneira dividia um referencial teórico comum, cujas raízes remontam à tradição neoinstitucionalista estadunidense,^3^ o corpo docente da sociologia parecia ter de encarar um desafio epistemológico um tanto mais árduo: coadunar uma formação especializada teórica e metodologicamente temática com as demandas mais amplas de partes organizadas de uma sociedade em transformação tanto social como politicamente.

A compreensão do que foi a geração pioneira na sociologia iuperjiana, contudo, não se limita à compreensão das suas trocas com a ciência política. Ponto igualmente relevante para tal entendimento está na variedade de laços político-intelectuais que aqueles sociólogos travaram com a própria disputa política. Esses cientistas sociais do Rio de Janeiro são tratados pela literatura especializada ora como excessivamente engajados na política, característica supostamente inercial dos isebianos (Toledo, 1977), ora como positivistas distantes da vida pública (Vianna et al., 1998), suposto efeito do americanismo próprio da formação dessa geração. Além de contraditórios entre si, esses desqualificativos não fazem jus à complexidade própria desses engajamentos. Primeiramente, como argumentamos alhures (Szwako, Araujo, 2019), o engajamento com atores políticos e da sociedade civil foi marca distintiva da ciência política uspiana liderada por Francisco Weffort. Mais ainda: o engajamento extrauniversitário com movimentos populares atravessou parcelas significativas da geração de cientistas sociais perseguidos e exonerados pós-1968 (Motta, 2014; Szwako, 2020). Assim, ao contrário do que tende a fazer crer parte da literatura sobre nossa história intelectual, a relação das e dos cientistas sociais com atores não acadêmicos (quer partidários, civis ou ambos) está longe de ser privilégio de intelectuais de instituições do Rio de Janeiro.

Como argumentaremos, mais comum parece ter sido o padrão de duplifiliação junto à carreira docente na sociologia iuperjiana. Aqueles jovens docentes penderam para dois tipos de atuação não excludentes: uma junto a agências estatais e da sociedade civil, em geral como especialistas e gestores, outra como assessores e consultores dos nossos emergentes movimentos sociais – movimento feminista, movimento negro, movimento por habitação etc. Portanto, a nosso ver, resta inadequada a ideia de que “uma das marcas da sociologia carioca seja, até o presente, um comprometimento político mais evidente do que o da sociologia paulista” (Jackson, 2007, p.117).

Se olhada superficialmente, a sociologia iuperjiana parece ter sido menos envolvida no debate político, especialmente, se contrastada com sua congênere paulista ou seu antecedente carioca, o Iseb. Nesse sentido, basta lembrar que, contrariando versões da autoimagem uspiana,^4^ Florestan Fernandes não só nutriu, quando jovem, laços partidários revolucionários, mas também se aproximou eventualmente das elites paulistas (ver Romao, 2006), tendo, já maduro, sido eleito e reeleito pelo mesmo Partido dos Trabalhadores, fundado, entre outras figuras, por Francisco Weffort, no começo dos anos 1980 – uma saga, enfim, repleta de apostas e aproximações com a política. Na trilha daquela ideia de filiação extra-acadêmica e em oposição a uma versão heroica de nossas ciências sociais, propomos observar a sociologia iuperjiana por lentes político-intelectuais suficientemente plásticas para incorporar a ambivalência da circulação de grupos e indivíduos entre pesquisa, militância e burocracias governamentais, sem recurso a noções inadequadas para o caso brasileiro, como é o caso, sobretudo, da noção de “sociologia pública” (Perlatto, Maia, 2012; Szwako, 2020).

Assim, nossa leitura da vida institucional e intelectual de uma parte sobejamente negligenciada do Iuperj visa mais que iluminar a ação desse conjunto de sociólogos. Na esteira de outros trabalhos (Szwako, Araujo, 2019), almeja também desamarrar a história das ciências sociais brasileiras do encapsulamento teórico imposto por noções que idealizam a conformação histórico-intelectual de nossas disciplinas, reduzindo-as a uma noção limitada e limitante como a de “campo” (Szwako, 2012). Fundamental nessa idealização é sua separação a fórceps entre ciência e política: enquanto do caso carioca faz-se uma caricatura, segundo a qual “[não] houve na então capital federal vida acadêmica propriamente dita” (Jackson, 2007, p.117), o projeto uspiano é desligado de suas determinações político-formais nas vezes em que as evidências “reforçam ... o entrelaçamento direto das ciências sociais [paulistas] com a política, típico desse ‘estado do campo’ não institucionalizado plenamente” (p.121).

Uma derivação mais elaborada dessa perspectiva é a tese de Keinert (2011). Para ele, o profissionalismo iuperjiano se desenvolveu em íntima relação com os anseios de uma geração no sentido de influenciar politicamente um Estado que mesclava autoritarismo com projetos modernizadores. O fator determinante de tal sucesso teria sido a “coesão entre setores da elite brasileira” que produziu lideranças personalistas como Fernando Henrique Cardoso no Centro Brasileiro de Análise e Planejamento (Cebrap), Roberto Cardoso de Oliveira no Museu Nacional, e Cândido Mendes no Iuperj (Keinert, 2011, p.5-6). De laços sociais tradicionais, teria emergido uma lógica institucional moderna que abriu espaço para uma classe média em ascensão educacional, mas ainda modesta em capital cultural para os padrões do período (p.9). A esta, restou a solução de buscar novos signos de legitimidade em especializações nos EUA e, depois disso, circular “por cargos de naturezas distintas, em associações científicas, agências de fomento à pesquisa, além da coordenação dos cursos pós-graduados”, bem como na “assessoria aos partidos e movimentos sociais, a partir de fins da década de 1970, até os postos burocráticos em órgãos governamentais, com a redemocratização do país em 1985” (p.7).

A análise de Keinert reitera não somente uma abordagem disposicional dos iuperjianos – na qual as estratégias de legitimação são deduzidas dos dilemas institucionais e das origens sociais da geração –, como também os paradoxos próprios da geração anterior no Iseb, já que a incerteza material teria feito com que “a esfera pública [permanecesse] sendo uma instância importante de legitimação” (Keinert, 2011, p.7). Apesar de pródigo em elucidar a emergência desse novo modelo de pós-graduação e jogar luz sobre as estratégias de legitimação dessas gêneses organizacionais, o diagnóstico de Keinert não é suficiente para explicar tudo que envolveu a sedimentação dessas instituições. Particularmente, a ênfase que elas colocavam na autonomia científica dos pós-graduandos, fosse na escolha de seus temas, na definição de suas metodologias ou ainda em sua pluralidade política. De acordo com o primeiro Caderno de Ementas (1971, p.2), “fundado numa perspectiva liberal da educação, o Programa de Mestrado busca promover a competência por meio do único critério de avaliação legítimo no universo acadêmico – a persuasão racional”.

Queremos aqui propor uma leitura alternativa a esse tipo de mirada sobre a institucionalização acadêmica de inspiração bourdieusiana que, no Brasil, teve nos dois volumes de Miceli (1995, 2001) seu principal ponto de irradiação. Dessa perspectiva, os projetos pedagógicos e científicos à raiz das ciências sociais são encarados como discursos arbitrários, mobilizados para eufemizar lutas por prestígio e legitimidade acadêmica, de grupos cujas práticas se explicam mais pelas suas trajetórias e disposições de classe do que por suas ideias ou propostas substantivas. Consideramos essa abordagem empiricamente limitada, pois insiste em reduzir sistemas acadêmicos altamente complexos e profundamente diferenciados a “espaços sociais de lutas”, sempre determinados por interesses e disputas orientados por disposições exteriores. Se esse modelo pode servir para explicar processos de gênese institucional acadêmica, seus rendimentos heurísticos decaem junto da complexificação das dinâmicas de estruturação e especialização de mundos acadêmicos maiores.^5^

Nosso interesse aqui é distinto. Mais do que desvelar os interesses extra-acadêmicos, supostamente escamoteados nos projetos pedagógicos em análise, pretendemos compreender a noção de vocação científica própria de uma geração intelectual. Isso não implica necessariamente uma mirada purista ou inocente desses projetos, que eventualmente podem por certo responder a interesses de naturezas diversas. Significa, antes, que a estruturação disciplinar e pedagógica assumida por uma disciplina/instituição (a sociologia do Iuperj, em nosso caso) não precisa ser resumida a tais interesses que, por sua vez, não conseguem explicar por que uma determinada concepção de vocação científica logrou imprimir-se institucionalmente.

Como iremos argumentar, por um lado, as apostas e propostas intelectuais (pedagógicas, curriculares, organizacionais, científicas) não são mecanicamente determinadas pelo conflito político nem pela trajetória social de seus precursores; tais apostas são, antes, mediadas por sua interação constante com representantes eleitos, burocracias, partidos, sistema de justiça, movimentos e associações da sociedade civil. Ainda em esboço, esse modelo de uma sociologia política da vida, a um só tempo, institucional e intelectual do antigo Iuperj pode, quem sabe, deslocar as amarras de nossa própria história intelectual.

Por outro lado, queremos sugerir a noção de “vocação” como alternativa heurística àquela história das ciências sociais distinguida pela ênfase em disposições e estratégias intelectuais centradas em disputas por espaço (ver Miceli, 2001). Como se sabe, foi Weber (1998) quem consagrou a noção de vocação – *beruf* – não apenas em suas preleções sobre ciência e política, mas também em sua sociologia da religião (Pierucci, 2005). Com ela, ele queria enfatizar não apenas a necessidade de produção histórica de um *ethos* próprio ao fazer científico, mas sua dependência em relação à especialização e à substituição de uma concepção missionária e religiosa que justificasse os pendores disciplinares. Diz ele:

Em verdade, os senhores esperam que eu lhes fale ... da vocação científica propriamente dita. Em nossos dias e referida à organização científica, essa vocação é determinada, antes de tudo, pelo fato de que a ciência atingiu um estágio de especialização que ela outrora não conhecia e no qual, ao que nos é dado julgar, se manterá para sempre. A afirmação tem sentido não apenas em relação às condições externas do trabalho científico, ... pois jamais um indivíduo poderá ter a certeza de alcançar qualquer coisa de verdadeiramente valioso no domínio da ciência, sem possuir uma rigorosa especialização. ... Só a especialização estrita permitirá que o trabalhador científico experimente por uma vez, e certamente não mais que por uma vez, a satisfação de dizer a si mesmo: desta vez, consegui algo que ‘permanecerá’. Em nosso tempo, obra verdadeiramente definitiva e importante é sempre obra de especialista (Weber, 1998, p.24; destaque no original).

Weber entende a ciência moderna em sua duplicidade: como prática profissional e simultaneamente como prática disciplinar (ver Dubois, 2016).^6^ Para dar conta dessa dupla dimensão, lançamos neste texto luz sobre os modelos organizacionais e suportes técnico-científicos que marcaram e demarcaram não campos autônomos institucionalizados, mas sim práticas, normas e conteúdos disciplinares – neste caso – da primeira geração de sociólogos do Iuperj. Dito de outra forma, embora a noção de campo possa ser valorosa quando se busca modelar conflitos por *status* em campos altamente institucionalizados e nos quais os agentes dividem uma libido e uma opinião basilares comuns – que Bourdieu chamava, respectivamente, de *illusio* e de *doxa* –, ela perde potencial heurístico quando o objetivo é compreender a emergência de novas vocações profissionais em campos pouco institucionalizados ou em vias de institucionalização. Nesses casos, mais que pressupor a cumplicidade ontológica entre pendores subjetivos e aparatos organizacionais, trata-se de entender como orientações disciplinares produziram estruturas organizacionais e, ao mesmo tempo, bússolas normativas para sua ação. É nesse sentido que a ideia de vocação pode, como sugerimos, constituir-se como instrumento analítico mais sensível ao processo de formação de normas práticas do que de sua reprodução.

## Iuperj: anos e perfis iniciais

O primeiro obstáculo a ser enfrentado aqui tem a ver justamente com a dificuldade em demarcar o que era esta sociologia do Iuperj e quem eram seus praticantes. Isso porque o instituto se caracterizou por recrutar pesquisadores de formação híbrida, lotados, muitas vezes, simultaneamente nos dois programas de pós-graduação. Fundado em 1963, no âmbito da Universidade Candido Mendes (Ucam), o Iuperj não foi inicialmente uma pós-graduação, mas um centro de pesquisa aplicada (Lynch, 2017). Era intenção da Fundação Ford incentivar a criação de especializações em ciência política no Brasil,^7^ o que levou Cândido Mendes, então reitor, a propor ao fundo a criação de um programa de mestrado nesse departamento da Ucam (Amorim, 2021, p.15).

A primeira geração de pesquisadores do centro foi, em parte, oriunda do Departamento de Sociologia e Política da UFMG, que já havia recebido financiamento da Ford para a criação de uma especialização em ciência política (Brockmann Machado, 1993; Amorim, 2021), e do já mencionado Iseb. Parte desses ex-isebianos foi convidada a cursar suas pós-graduações em ciência política nos EUA sob os auspícios da Ford; nomeadamente, Wanderley Guilherme dos Santos, Carlos Estevam Martins e César Guimarães. A parte dos precursores oriunda da UFMG, e que já possuía doutorado, notadamente Bolívar Lamounier, fundou o programa de mestrado em ciência política do instituto em 1969. O caráter privado do Iuperj facilitava a gestão de recursos da Ford, ao mesmo tempo que permitia a precarização do vínculo profissional dos pesquisadores. Talvez por conta disso, Lamounier se demite do instituto em 1971, migrando para seu análogo paulista, o Cebrap. Esse período coincide com o retorno daqueles que haviam viajado para cursar pós-graduação nos EUA.

Esse momento, no entanto, não foi feito só de politólogos. Já em seus anos iniciais, sociólogos se somaram à equipe do antigo Iuperj: Neuma Aguiar, graduada em história pela Pontifícia Universidade Católica do Rio de Janeiro (PUC-Rio) e doutora em sociologia pela Washington University, em 1969; o argentino Carlos Alfredo Hasenbalg, mestre em sociologia pela Faculdade Latino-Americana de Ciências Sociais (Flacso), desde 1968; e o colombiano Fernando Uricoechea, especializado em sociologia pela London School of Economics, também em 1968. Todos compuseram o Programa de Mestrado em Ciência Política e Sociologia do Iuperj até novembro de 1972, quando os dois programas se separaram formalmente. As disciplinas de sociologia começam, então, a ser ofertadas efetivamente em 1973 (Caderno de Ementas..., 1973).

Ao que tudo indica, as razões para a separação foram de ordem burocrática: além de melhor abrigar a metade do corpo docente oriunda da sociologia, a duplicação aumentava as chances de obtenção de recursos das fundações privadas e, sobretudo, públicas (Caminhos..., 4 jul. 2020, 14 nov. 2019). Embora os investimentos da Fundação Ford tenham sido centrais na viabilização do Iuperj (Amorim, 2021), a Coordenação de Aperfeiçoamento de Pessoal de Nível Superior (Capes) e, sobretudo, a Financiadora de Estudos e Projetos (Finep) (Chaves, 2018; Keinert, 2011) reorientaram recursos vultosos para o instituto na década de 1970, inclusive com assessoramento de alguns de seus pesquisadores e ex-pesquisadores como Simon Schwartzman e Mario Brockman Machado (1993). De todo modo, os dois programas mantiveram relações próximas em muitos níveis, haja vista que a ciência política foi incluída como área conexa de formação na sociologia, e vice-versa. Na prática, professores dos dois programas podiam orientar alunos de ambos, um rol de disciplinas era comum, e todos cooperavam em pesquisas compartilhadas (Caderno de Ementas..., 1973).

A Neuma Aguiar é reputada a liderança no processo de composição do programa de sociologia, ao qual se agregaram em 1973 Luiz Antônio Machado da Silva, então mestre em antropologia social pelo Museu Nacional em 1971, e Edmundo Campos Coelho, que acabava de concluir seu mestrado em sociologia na Universidade da Califórnia. A partir daí, haveria uma reorganização do corpo docente. Simon Schwartzman foi alocado no Programa de Sociologia, junto também com Carlos Hasenbalg e Nelson do Valle Silva, que já lecionava cursos desde 1971, mesmo tendo formação em economia e mestrado em informática.

A reunião desses nomes já sugere uma diversidade temática, formativa e de abordagem. Neuma Aguiar foi inicialmente uma socióloga do desenvolvimento industrial, mas que paulatinamente migrou rumo às pesquisas sobre as desigualdades de gênero e é hoje considerada uma precursora desta área na sociologia brasileira. Perfil similar ao do argentino Carlos Hasenbalg, inicialmente estudioso das relações de classe nos processos de desenvolvimento, posteriormente interessado nos estudos de estratificação racial e educação, muitos deles redigidos em parceria com Nelson do Valle Silva (Osório, 2008). O peso do enquadramento teórico do desenvolvimento sobre essa geração fica nítido, especialmente, embora não exclusivamente, na produção de Hasenbalg. Apesar de, ao longo de sua obra, ter se dedicado fundamentalmente a escrutinar os mecanismos de reprodução das desigualdades raciais no Brasil, tais mecanismos são trazidos à baila para questionar as teorias da modernização em voga, que, *à la* Florestan Fernandes, atribuíam certa autonomia do desenvolvimento capitalista perante o racismo. Na equação de Hasenbalg (1979), a quadra histórica de uma “sociedade competitiva” não corresponderia, nem levaria *per se*, a mais igualdade em termos raciais.

Outro componente dessa geração pioneira foi Luiz Antonio Machado da Silva, conhecido pelos trabalhos sobre o mercado de trabalho informal, tendo posteriormente incorporado distintas temáticas como sociologia urbana dos movimentos sociais, da criminalidade e da violência. Também preocupado inicialmente com as teorias do desenvolvimento, sobretudo com a aplicação ao mundo do trabalho da noção de “marginalidade estrutural”, Machado da Silva foi um dos precursores do conceito alternativo de informalidade, até hoje central na leitura de nossas desigualdades (Machado da Silva et al., 2018). Ele também foi percursor nos estudos de habitação e favelas, tendo participado diretamente dos então chamados movimentos de favelados.

Simon Schwartzman e Fernando Uricoechea, por seu turno, foram estudiosos do processo de formação do Estado nacional e de sua modernização no Brasil. O leque de objetos de estudos de Schwartzman, no entanto, vai além da formação nacional, indo desde o autoritarismo brasileiro até a formação de nossa ciência e nosso sistema de ensino superior, para citar apenas alguns de seus temas de maior influência (ver Schwartzman, 2008). Finalmente, Edmundo Campos Coelho, que se dedicou à pesquisa de diferentes tipos de organizações sociais, de prisões à própria academia, passando pela sociologia das profissões.

Para além das diferenças, há similaridades e convergências entre esses sete nomes, especialmente em termos de formação. A primeira delas tem a ver com suas graduações. Foram basicamente três os centros de formação inicial dessa geração: o curso de sociologia política da PUC-Rio, onde se formou Luiz Antonio Machado, mas com o qual tiveram contato estreito Neuma Aguiar e Nelson do Valle; a Faculdade de Sociologia e Política da UFMG, centro pioneiro da ciência política e onde se formaram Simon Schwartzman e Edmundo Campos; e a Flacso do Chile, então dirigida por outro colaborador do instituto, Glaucio Ary Dillon Soares, e onde se formou Carlos Hasenbalg. Glaucio Soares foi, inclusive, um dos responsáveis pela formação metodológica de Neuma Aguiar e de sua consequente ida para os EUA (Caminhos..., 4 jul. 2020). Essas sobreposições de trajetórias trazem um traço distintivo comum: a origem estadunidense da formação pós-graduada dessa geração pioneira da sociologia iuperjiana. Quer dizer, todos esses nomes tiveram mestrado ou doutorado, ou ambos, em grandes universidades dos EUA na década de 1970. Tal origem, diga-se de passagem, parece um tanto heterodoxa em relação aos departamentos de sociologia então ativos no país, majoritariamente orientados pelo e para o continente europeu. Além dessa formação comum, esse conjunto de docentes compartilhava de projeto científico-pedagógico de cunho especializante. É o que vamos ver no tópico a seguir.

## Modelo organizacional e a tensão totalidade/especialização

No mínimo desde 1971, o Iuperj oferecia cursos metodológicos e temáticos no sistema de créditos. Como é sabido, antes da reforma universitária de 1968, a pós-graduação no Brasil se baseava em um sistema tutorial francês, no qual os alunos tinham uma formação ligada às diretrizes dos professores catedráticos e cursavam pouquíssimos seminários, quase todos sem ementas ou regras previamente formalizadas (Pulici, 2007). No contexto pré-reforma universitária, o foco recaía menos na formação ou em um treinamento plural, e mais na dedicação à produção supervisionada de uma monografia.

No Iuperj, por sua vez, os alunos tinham de completar uma grade básica de cursos obrigatórios, mormente teóricos e metodológicos, além de cursos temáticos optativos de livre escolha, todos com bibliografia e regras previamente definidas. Nesse modelo organizacional, a figura do professor catedrático hegemônico cedia lugar ao professor orientador, uma espécie de conselheiro escolhido pelo próprio estudante no segundo ano do mestrado. Vale lembrar que, no sistema catedrático, muitas das escolhas de pesquisa relacionadas aos métodos, aos cursos ou mesmo aos temas a ser estudados ficavam nas mãos do professor catedrático.^8^ Já no sistema iuperjiano, os cursos, métodos e temas das dissertações eram definidos pelos matriculados, como indicava já o primeiro caderno de ementas, “sendo os alunos altamente estimulados a exercerem sem reservas seu espírito crítico, assim como a desenvolverem os estudos que considerarem mais adequados à sua inclinação intelectual” (Caderno de Ementas..., 1971, p.2).

Da perspectiva do corpo discente, o curso focalizava o treinamento de pesquisadores especializados. Da perspectiva do corpo docente, os professores não mais se organizavam hierarquicamente em torno de uma cátedra, tornando-se pares em uma estrutura administrativa que incentivava o desenvolvimento de agendas paralelas. Isso já aparece no parágrafo introdutório do Caderno de Ementas de 1971:

A execução do programa de mestrado baseia-se na atividade docente e de pesquisa, de um corpo de cientistas políticos de comprovada competência profissional e permanente interesse na formação de novos investigadores. Ademais de oferecer a maior variedade possível de cursos à escolha dos alunos, procura o programa manter como norma de formação, o pluralismo intelectual indispensável à constituição de uma atitude científica não paroquial. Para o programa não existem escolas privilegiadas nem verdades intocáveis ... Fundado numa perspectiva liberal da educação, o Programa de Mestrado busca promover a competência por meio do único critério de avaliação legítimo no universo acadêmico – a persuasão racional (Caderno de Ementas..., 1971, p.2).

Esse trecho faz um acerto de contas com a herança isebiana e seu legado nacionalista, agora criticado, pois “não existem verdades intocáveis”. Por outro lado, e orientada para outra audiência, a autonomeação “liberal” não diz respeito somente a uma filiação normativo-ideológica, ainda que parte de seus docentes fundadores tivesse tal filiação. A nosso ver, “liberal” aí diz respeito à imagem que o Iuperj queria passar aos públicos e aparelhos repressivos que, mesmo em se tratando de um ente privado, seguiu vigiando de perto as atividades do Instituto.^9^

Dentro do funcionamento organizacional, as propostas de curso passam, pelo menos desde 1971, a ser submetidas ao corpo docente fixo, e, uma vez aprovadas, eram editadas no “caderno de ementas” disponibilizado aos alunos. Tendo como norma o “pluralismo intelectual”, defendia-se o compromisso com um programa de curso que oferecesse um rol de disciplinas capaz de possibilitar aos estudantes que desenvolvam autonomamente seu “espírito crítico”. Tal defesa de especialização volta, pouco adiante, a ser enfatizada com a fundação do mestrado em sociologia:

O Mestrado em Sociologia do Instituto Universitário de Pesquisas do Rio de Janeiro foi criado em novembro de 1972. A finalidade deste curso é formar pessoas que executem trabalhos originais e independentes de pesquisa em Sociologia. O programa de cursos está voltado para o estudo dos temas: Estrutura e Mudança Social, muito embora a perspectiva do programa seja a de não especializar os estudantes demasiadamente em uma determinada área da Sociologia, procurando fornecer-lhes os instrumentos conceituais e técnicos que permita-lhes desenvolver seus próprios campos de investigação limitados apenas pela capacidade de oferecer conhecimentos de que dispõe o curso (Caderno de Ementas..., 1973, s.p.).

Vale notar que o equilíbrio dessa especialização não era só a aplicação de um modelo estadunidense, mas também objeto de reflexão teórica. Muitos dos professores supramencionados se dedicaram a pensar a agenda do desenvolvimento nacional, hegemônica no Brasil até meados dos anos 1970 (Leme, 2015). Cada um e cada uma, no entanto, problematizam esse compromisso com a totalidade com as tensões impostas pela especialização temática, teórica e metodológica.

Apesar de ser hoje ponto relativamente pacífico, a busca por uma especialização foi alvo de controvérsia desde os primeiros anos do Iuperj. A despeito das diferenças de perspectiva, a especialização temática e metodológica era vista com cautela e receio. Pista expressiva dessa tensão ficou impressa no primeiro editorial da revista *Dados* (Tema, 1966). Nele, a cautela diante da especialização carrega uma preocupação com a “ação concreta”, evocando certa nostalgia da figura do “intelectual público” (ver Lovatto, 2021, p.11), tão cara ao extinto Iseb:

A retomada que pretendemos da procura da totalidade, reenceta um estilo ensaiado, por exemplo, há uma década, por *Cadernos do Nosso Tempo*. Esta publicação foi responsável, mais que qualquer outra, pelo maior número de hipóteses criadoras que converteram o nosso contínuo social à sua peculiaridade, e deram início a um legítimo esforço de compreensão da realidade brasileira ... Mas quer-se dedicar ao esforço de rigorosa precisão no seu entendimento ... reduzindo e purgando o conteúdo ideológico de todo um arsenal de categorias de investigação da realidade brasileira, forjados de imediato para uma ação concreta, desbordados, sôfregos, do plano científico para o da ação das inteligências (Tema, 1966, p.4-5).

Analogamente, a atenção a uma ideia de totalidade, bem como a cautela com a especialização reaparecem, ambas, nos primeiros documentos institucionais da área da sociologia iuperjiana. Como vimos, aquele Caderno de Ementas (1973, s.p.) do mestrado estava preocupado em “não especializar os estudantes demasiadamente em uma determinada área da Sociologia”.

Essa, contudo, não era apenas uma questão pedagógica. Ela atravessa a agenda teórica de parte daqueles professores. Nas páginas de *Dados*, Simon Schwartzman (1971, p.30), por exemplo, propôs uma reformulação dos termos do debate ao redor do par totalidade/especialização.^10^ Contra a ideia de uma eterna juventude essencial às nossas ciências sociais, ele afirmava que “[uma] ênfase excessiva em teorização de alcance médio e uma repressão prematura de atividade podem significar a proliferação de pesquisas esparsas que têm pouca relevância, como também, e o que é pior, não apresentam cumulatividade”. Sua aposta teórico-pedagógica propunha enquadrar as pesquisas das ciências sociais entre Kuhn e Merton, meio lá meio cá, orientando-as para um ponto intermediário entre especialização e totalidade:

Tudo indica que não se chegará a uma nova abordagem dos fenômenos sociais, ou um novo paradigma, mas a uma pluralidade de linhas de pesquisa e desenvolvimento teórico simultâneos, muitas vezes complementares, mas também muitas vezes não relacionados ou incompatíveis ... Teorias de desenvolvimento nacional e internacional podem ser derivadas das características do sistema internacional, com a ajuda de indução estatística sistemática; teorias de jogos e de processos decisórios são usadas para a predição de fenômenos de curta duração, tipologias internacionais são desenvolvidas, e modelos computarizados de sociedades são experimentados (Schwartzman, 1971, p.42-43).

O debate sobre especialização perpassava outros institutos do período, mas o diferencial do Iuperj nesse momento era a sua tradução em critérios organizacionais próprios. Como veremos a seguir, essa tensão entre especialização e totalidade se resolveu, na prática pedagógica, por meio de um currículo que oferecesse uma pluralidade de temas e metodologias aos jovens cientistas em treinamento, sem, contudo, perder de vista a preocupação com “ação concreta”, ou seja, com movimentos e engajamentos extra-acadêmicos. Ademais, o empreendimento dessa sociologia especializada não foi apenas, nem, sobretudo, reflexo da influência dos diplomas estadunidenses acumulados no Iuperj. Tratou-se, antes, da forma intelectual pela qual os desafios do contexto nacional de então foram lidos, incorporados e traduzidos na agenda da sociologia iuperjiana.

## Uma sociologia política especializada: o currículo

A gênese da sociologia iuperjiana está atravessada em múltiplos sentidos pela convivência com o programa de ciência política. Em um sentido primeiro e mais trivial, essa influência é recíproca, tal como se pode ver no seguinte trecho: “O Mestrado em Sociologia possui como área de domínio conexo a Ciência Política, constituindo, por sua vez, área de domínio conexo para a área de Ciência Política” (Caderno de Ementas..., 1973, s.p.). Para além dessa marca genética, a sociologia do Iuperj nasceu também das trocas com outros programas de pós-graduação, com os quais mantinha “convênio informal” (Caderno de Ementas..., 1973), tal como era o caso do Museu Nacional e da Pós-graduação em Engenharia da Universidade Federal do Rio de Janeiro (Keinert, 2011).

A interlocução com a ciência política fica expressa também no elenco de seminários e de disciplinas oferecidas entre 1973 e 1977. Os títulos dos cursos oferecidos falam bastante do hibridismo disciplinar do mestrado então nascente. Considerando o período analisado, foram ministrados ou previstos os seguintes cursos: “Sociologia política das sociedades industriais”, “Estado e sociedade”, “Sociologia do desenvolvimento”, “Seminários de mudança social: alternativas do desenvolvimento” (Caderno de Ementas..., 1973); “Sociologia do desenvolvimento”, “Dominação e administração”, “Sociologia política das organizações” (Caderno de Ementas..., 1974); “Estruturas públicas e sociais no Brasil”, “Estrutura social da América Latina”, “Estrutura ocupacional do Brasil” (Caderno de Ementas..., 1975); “Sociologia política comparada”, “Estratificação social”, “Educação e desenvolvimento” (Caderno de Ementas..., 1976). Para 1977, o leque de cursos ministrados reproduz essa interlocução sociopolitológica, ao mesmo tempo que a preocupação com a agenda do desenvolvimento parece perder peso na grade curricular: “Teoria social e ordem política”, “Política urbana”, “Mudança social”, “Estratificação e classes sociais” (Caderno de Ementas..., 1977).

Distante da estadofobia cara a outras latitudes (ver Szwako, Araujo, 2019), esse diapasão maior de uma “sociologia política” era fortemente atravessado pelo “problema clássico das relações entre estado e sociedade”, tal como foi o caso da “Sociologia política das organizações” (Caderno de Ementas..., 1974, s.p.). Nesse mesmo sentido, e não por acaso, “Estado e sociedade” é o nome de uma optativa que vai marcar longamente não só a tradição iuperjiana, mas também seus herdeiros contemporâneos.^11^

No plano curricular, a especialização se traduz na oferta de disciplinas temáticas que, no conjunto de todas as disciplinas ofertadas, são a maioria. Se considerarmos todas as disciplinas oferecidas ao corpo discente do Mestrado de Sociologia, entre 1973 e 1977, teremos 55 disciplinas desse tipo – aí considerada a soma daqueles cursos ofertados à sociologia com aqueles ofertados a ambos, sociologia e ciência política. Além dessas, eram regularmente oferecidas disciplinas de teoria sociológica (16 vezes); metodologia quantitativa (18 vezes) e seminário (oito vezes).

Ao mesmo tempo que é vocacionada por aquela sociologia política, a oferta das disciplinas temáticas abrange um rol de temas de alcance expressivo: indo de “Sociologia da saúde mental”, “Educação e desenvolvimento” e “Sociologia da família”, passando pelos “Temas de sociologia da ciência e política científica” e pela “Economia política do desenvolvimento”, chegando a “Marginalidade” e, ainda, a “Estratificação e estrutura ocupacional na cidade do Rio de Janeiro na segunda metade do século XIX” – mesmo utilizando-se de uma parcela abreviada desse leque curricular, é possível notar a variedade de conteúdos então disponíveis para a especialização na formação iuperjiana.

Outros dois pontos nevrálgicos desse currículo são as disciplinas de “teoria” e de “metodologia”. Em certa medida, a marca daquilo que seria próprio à sociologia, em relação de distinção quanto à ciência política, pode ser procurada e observada no oferecimento dos cursos obrigatórios – ou “requisitados”, como eram na época chamados – de “Teoria sociológica”. Neles, a consagração do cânone do século XIX é tanto alternada como posta em interlocução com a produção “contemporânea”, isto é, com aquilo que era considerado *up to date* nos anos 1970. Enquanto “fazer uma análise das contribuições de Durkheim” (Caderno de Ementas..., 1973, s.p.) era o maior objetivo de uma disciplina teórica em 1973, a disciplina teórica do ano seguinte se dedica às “obras de Weber”, então “pouco estudadas nos cursos de graduação em ciências sociais”, para ir além dele, vendo o alemão “como precursor dos trabalhos recentes que vêm sendo desenvolvidos no campo da etnometodologia” (Caderno de Ementas..., 1974, s.p.). Já no segundo semestre de 1974, vemos um curso mais eclético oferecido por Wanderley Guilherme dos Santos a ambas, sociologia e ciência política, qual seja, “Teoria social: política contemporânea”, que visava “tornar familiar aos estudantes os textos que apresentam algumas das abordagens contemporâneas aos fenômenos políticos”, começando por Merton e Parsons, para daí deslindar uma tradição estadunidense de teóricos “clássicos, por assim dizer, pelo menos por ora” (Caderno de Ementas..., 1974, s.p.).

Seguindo uma tendência mais ampla, a oferta da disciplina teórica parece, entre 1975 e 1977, ter ganhado relativa estabilização quanto a quem seria parte dos “clássicos”. “O curso cobrirá as principais contribuições de Marx, Weber e Durkheim” (Caderno de Ementas..., 1975, s.p.). No ano seguinte, “[o] curso visa a explorar diferentes interpretações de textos da sociologia clássica ... Todos os textos selecionados de autores clássicos serão obrigatoriamente lidos (Durkheim, Mauss, Weber, Marx e Simmel)” (Caderno de Ementas..., 1976, s.p.). Já em 1977, se pretende “dar uma visão do debate entre várias correntes sociológicas atuais (etnometodologia, interação simbólica, fenomenologia sociológica, marxismo crítico, marxismo estruturalista e ... estruturalismo), procurando mostrar as raízes do debate contemporâneo na teoria clássica”, entendida então em função de “três tradições: durkheimiana, a weberiana e a marxista” (Caderno de Ementas..., 1977, s.p.). Como sugerimos, essa disposição curricular inseria o Iuperj em uma tendência internacional maior marcada pelo processo de invenção do cânone sociológico. Pois, ao contrário do que se pode e se tende a pensar, os “clássicos” do século XIX (nomeadamente, Marx, Weber e Durkheim) tiveram sua consagração como “cânone”, na academia anglo-saxã, apenas no pós-guerra (Connell, 2020). Nos cursos teóricos da sociologia iuperjiana não foi diferente, embora, nas décadas seguintes de 1980 e 1990, os nomes de Simmel e Tocqueville tenham, com a saída de Mauss, se alternado ao lado do panteão desde então canonizado.

Por fim, na distribuição curricular da sociologia iuperjiana, cabe também a composição das disciplinas dedicadas a “metodologia”. No conjunto de disciplinas especificamente metodológicas ofertadas, 14 delas foram “quantitativas”, quatro “qualitativas” (ou “não quantitativas”, como também foram nomeadas), sendo apenas uma delas simultaneamente “qualitativa e quantitativa”. A prevalência observada no oferecimento dos métodos quantitativos não espelha somente a relação com a ciência política, pois o mestrado em sociologia contava com pesquisas e pesquisadores de estatísticas e estratificação social, desde sempre afeitos àqueles métodos. Seja como for, a proximidade com procedimentos quantitativos não era encarada como uma questão de afastamento ou exclusão. Ao contrário, nos cursos que visavam à “familiarização com técnicas de pesquisa não quantitativas – história de vida, observação participante e semiparticipante”, também se almejava uma “comparação entre elas e técnicas quantitativas” (Caderno de Ementas..., 1974, s.p.). No mesmo sentido, no curso de 1977, o objetivo era introduzir discentes nas “chamadas metodologias ‘qualitativas’ e ‘quantitativas’, não como corpo de procedimentos excludentes, mas complementares” (Caderno de Ementas..., 1977, s.p.); visava assim lançar luz sobre as “inúmeras divergências que se escondem na dicotomia ‘metodologia qualitativa – metodologia quantitativa’” (s.p.). Esssa era, aliás, outra distinção em relação ao programa de ciência política, na qual os cursos de metodologia qualitativa eram quase inexistentes.

A sociologia iuperjiana, assim observada, é dificilmente resumível à relação com sua irmã mais velha, a ciência política, embora tenha dela herdado parte central de sua vocação para uma sociologia política. Ora, esse qualificativo estampado nos títulos de tantas disciplinas está repleto de consequências para a concepção de prática de pesquisa, em especial, dentro de um instituto marcado pelo passado isebiano e, por isso, continuamente preocupado em não se divorciar da “ação concreta”. Em tais termos, o significado dessa vocação não depende apenas dos termos da reprodução pedagógico-curricular do projeto iuperjiano de uma formação especializada. O que tal vocação significou, na prática, depende também do repertório de pesquisas e relações então desenvolvidas por seus praticantes. Para finalizar, então, passemos aos laços acadêmicos e extra-acadêmicos forjados nas pesquisas desse conjunto pioneiro vocacionado para uma sociologia política compartilhada e simultaneamente especializada.

## Engajamentos extra-acadêmicos: burocracias estatais e sociedade civil

Diferentemente daqueles que, a exemplo de Arruda,^12^ buscam ver na história das ciências sociais brasileiras a constituição de “campos” institucionalizados (i.e., autonomizados), nossa observação do caso da sociologia iuperjiana escolhe lançar luz sobre a circulação de seus docentes, não só entre domínios disciplinares, mas também, e mais especialmente, entre esferas de atuação e interlocução – acadêmica, governamental, civil, partidária ou outra. Em sentido inverso ao das leituras em nota, vejamos neste tópico a vocação desses pesquisadores por meio de seus engajamentos: espremida entre agências de financiamento e agências repressivas, a sociologia iuperjiana engajou-se em outros espaços e com outros atores, na universidade e fora dela, quer no Brasil ou fora dele.

Além do crescimento e recrudescimento das agências e formas de repressão estatal, o regime político inaugurado em 1964 foi marcado também por uma complexificação *sui generis* das estruturas burocráticas. Para termos ideia desse crescimento, “60% das empresas públicas, fundações, autarquias e empresas estatais existentes [em 1995] foram criadas entre 1966 e 1976” (Martins, 1995, p.11). Ao mesmo tempo que ocorreu em vários níveis federativos, a criação de agências governamentais e paraestatais se deu com variados graus de autonomia e mesmo de excelência. Se a atuação dessas agências era monitorada de perto pelas forças repressoras, elas próprias organizadas em instituições como o Departamento de Ordem Política e Social (Dops), boa parte delas conquistou também alguma autonomia relativa.

Assim, os governos militares foram altamente ambíguos em relação às ciências. Na síntese de Ridenti (2014, p.36), tal “ambiguidade explica-se, em parte, porque a modernização exigia profissionais capacitados, muitos deles de oposição. ... Alguns professores incômodos eram afastados, mas a pesquisa e a tecnologia foram financiadas até no meio universitário mais avesso ao regime”. Isso significou um relativo paradoxo para as e os cientistas sociais vistos como “comunistas”. De um lado, do ponto de vista da política científica dos governos militares, agências criadas antes mesmo de 1964, como o Conselho Nacional de Desenvolvimento Científico e Tecnológico (CNPq) e a Capes, passaram a se conformar no período ditatorial como instâncias centrais na profissionalização e na institucionalização do fomento às nossas prática e comunidade científicas. Não é, então, por acaso que Schwartzman (2001) vê o período pós-1968 como um “grande salto” em nossa política científica. Contudo, de outro lado, em grande medida devido à vigilância imposta aos quadros das faculdades de filosofia que permaneceram na universidade mesmo após os expurgos pós-1968 (ver Motta, 2014), as ciências humanas foram o sócio menor e temporão nas formas e fontes de financiamento da pesquisa cientifica no país.

Seguindo esse padrão, as ciências sociais passaram a acessar, de maneira sistemática e paulatina, cifras e verbas vindas dessas duas agências apenas a partir do final da década de 1970 e em volume consideravelmente menor que as demais áreas do conhecimento. No entanto, à diferença de Capes e CNPq, outras agências internacionais e mesmo estatais, a exemplo, sobretudo, da Fundação Ford e da Finep, desempenharam função incontornável na “constituição da atual comunidade de cientistas sociais brasileiros” (Figueiredo, 1988, p.38).

Cientistas sociais taxados de “comunistas” não foram apenas perseguidos, tal como foi o caso do Iuperj inclusive; ao longo da década de 1970, parte deles também passou a compor as crescentes institucionalidades de fomento à ciência nacional. Foi, então, nessa configuração política fundamentalmente ambígua, marcada pelo crescimento de instâncias de regulação estatal e simultaneamente pela sombra da repressão, que circularam os docentes do Iuperj.

Simon Schwartzman, por exemplo, foi figura-chave na análise e na posterior formulação da política de fomento às ciências no Brasil, seara na qual já atuava seu colega da ciência política Mário Brockman Machado. Simon teve especial importância para a sociologia da ciência e da educação no Brasil, tendo-se tornado inúmeras vezes consultor em processos de formulação de políticas públicas para essas áreas em órgãos como CNPq e o Inep (Schwartzman, 2021). Já Nelson do Valle Silva foi funcionário de carreira do Instituto Brasileiro de Geografia e Estatística (IBGE) e, posteriormente, tornou-se um dos fundadores do Laboratório Nacional de Computação Científica do CNPq. Luiz Antônio Machado da Silva, por sua vez, tinha um histórico de pesquisas aplicadas, como aquelas sobre moradia que foram financiadas pelo Banco Nacional de Habitação.

Outra pista dos laços com órgãos estatais e paraestatais pode ser vista no relatório de gestão de dez anos do instituto, publicado por sua diretoria em 1976. Nele constavam 51 projetos coletivos que envolviam trinta instituições, cuja maioria era formada por órgãos governamentais e sociedade civil: Banco Interamericano de Desenvolvimento, Centro Brasileiro de Assistência Gerencial à Pequena e Média Empresa, Empresa Brasileira de Turismo, Conselho de Desenvolvimento das Comunidades da Companhia Progresso do Estado da Guanabara, Ministério do Interior; Superintendência do Desenvolvimento do Nordeste, Organização das Nações Unidas para Educação, Ciência e Cultura etc. Isso permitiu que muitos dos pesquisadores do Iuperj, alguns até com passado de militância política e impedidos de exercer funções públicas, construíssem vínculos duradouros com diversas agências do Estado.

Essa rede de apoio às pesquisas coletivas reproduzia uma lógica estadunidense de financiamento universitário, mas não apenas isso. Ela servia a outros dois fins complementares. O primeiro obedecia a uma lógica simultaneamente material e institucional, visando não só à estabilização institucional, mas também à remuneração dos pesquisadores, na época com vínculo precário com a Ucam. Foi só no final dos anos 1970 que a universidade registrou as carteiras de seus docentes e uniformizou a carreira, bem como seus salários. Esse foi um problema crônico que acompanhou toda a história do instituto e que se agravou nos anos 1990 e 2000, quando seu corpo docente e discente migrou para a Universidade do Estado do Rio de Janeiro e fundou o seu atual Instituto de Estudos Sociais e Políticos (Iesp-Uerj).

Outro fim dizia respeito à formação em pesquisa. Quer dizer, esses laços e convênios não forneciam apenas os recursos necessários para a manutenção do instituto, mas, simultaneamente, criavam oportunidades para o treinamento em pesquisa aplicada dos seus discentes. Nesse sentido, merece menção a criação no Iuperj de uma diretoria encarregada de articular esses projetos, dirigida por um aluno à época, Édson Nunes, e que chegou a empregar uma quantidade expressiva de discentes na instituição.

Essa circulação de acadêmicos, contudo, não se deu exclusivamente rumo ao Estado. Neuma Aguiar foi figura-chave em vários congressos feministas nacionais e internacionais. Carlos Hasenbalg e Nelson do Valle Silva cooperaram ativamente com organizações do movimento negro, tendo o primeiro se tornado um dos diretores do Centro de Estudos Afro-Asiaticos, órgão ligado à Ucam que articulou acadêmicos, militantes e agentes internacionais na década de 1980. Hasenbalg, aliás, chegou a publicar um livro em parceria com a socióloga e militante negra Lélia Gonzalez em 1981. Em parceria com a demógrafa Elza Berquó, então na Universidade de São Paulo e no Cebrap, Nelson também foi articulador-chave da incorporação dos movimentos feminista e negro nas discussões sobre indicadores sociais no IBGE. Analogamente, Luiz Antônio Machado da Silva também teve atuação de destaque no movimento de favelados por saneamento e melhores condições de habitação.

O comentário *ex post* de uma das herdeiras dessa geração de sociólogos iuperjianos, Elisa Reis, lança luz sobre a aproximação deles a setores e movimentos sociais, sintetizando a mediação que, a nosso ver, tendia a diferenciar politólogos de sociólogos: “Da perspectiva da sociologia”, diz ela, “há conexões muito íntimas com a vida política nacional, mas a identificação temática é menos óbvia. Acho ainda que é preciso pensar a questão não apenas da perspectiva da oferta [acadêmica], mas também da perspectiva da demanda de pesquisa. Muitos dos temas da sociologia surgiram em resposta a uma demanda” (Reis et al., 1997), isto é, em resposta a demandas tanto do Estado como da sociedade civil.

## Considerações finais

A primeira geração da sociologia do Iuperj se unia por suas diferenças, isto é, por uma concepção da vocação sociológica fortemente centrada na ideia de especialização temática e metodológica. Todos eram incentivadores de uma disciplina especializada, tematicamente diversa, metodologicamente profissionalizada e orientada para a pesquisa empírica. Logo, eram cientistas sociais que convergiam no elogio à pluralidade de abordagens e se opunham à sociologia das grandes sínteses ensaísticas sobre o Brasil. Eram também contrários a uma sociologia especializada, porém, internamente estratificada, como eram as propostas presentes em vocações outras. É bem verdade que muitos dos nomes que marcaram essa história produziram teses que se somaram ao cânone de trabalhos sobre a modernidade brasileira. Em todos esses casos, a pesquisa empírica não era vista como uma etapa preparatória para uma síntese teórica, mas como parte de um trabalho acadêmico maior e mais complexo.

Ainda nas décadas de 1970 e 1980, somaram-se ao corpo docente nomes como Elisa Reis, Luiz Werneck-Vianna e Lícia Valladares. Embora eles tenham agregado novas perspectivas, todos haviam se formado em cursos do instituto e/ou atuado em pesquisas no seu interior. Agregaram-se, portanto, a um modelo organizacional consolidado a partir de uma concepção de sociologia política, como vimos, calcada na especialização temática, teórica e metodológica. Com as devidas proporções, o mesmo parece valer para os pesquisadores posteriormente incorporados: Ricardo Benzaquen de Araújo, Maria Alice Rezende, Adalberto Cardoso, José Maurício Domingues etc.

Estudos ulteriores poderão iluminar melhor a influência desse modelo disciplinar na organização da pós-graduação atualmente existente no Brasil, seja por meio das políticas científicas adotadas por agências como CNPq e Capes, seja com base nas normas internamente gestadas a partir da Associação Nacional de Pós-graduação e Pesquisa em Ciências Sociais, associação fundada no âmbito do próprio Iuperj. Como hipótese, porém, pode-se sugerir que o instituto pode ter sido um centro irradiador do modelo adotado pela reforma universitária de 1969 e até hoje hegemônico no Brasil. Esse modelo não se resumia apenas aos cursos distribuídos em créditos ou à redução dos poderes acadêmicos dos catedráticos, mas também a uma ênfase nas pesquisas aplicadas coletivas e nas cooperações com o Estado e a sociedade.

Apesar disso, se o estatuto privado da sociologia iuperjiana representou um diferencial de sua pós-graduação no período ditatorial, a redemocratização do Estado brasileiro ameaçou sua subsistência. A difusão do seu modelo de pós-graduação criou em todo o país uma concorrência não apenas acadêmica, mas também pelas verbas públicas e privadas de pesquisa, cada vez mais escassas. Essa crise encontrou seu cume em 2011, quando, depois de meses sem receber os salários da Ucam, o corpo docente e discente do Iuperj migrou para a Uerj e fundou em seu interior o Iesp.

De todo modo, não foi nosso objetivo aqui avaliar a continuidade desse modelo disciplinar no atual Iesp-Uerj ou na pós-graduação brasileira de modo geral. Nosso intento foi mais modesto: avaliar se havia uma agenda, tanto epistemológica como política, na primeira geração de sociólogos do Iuperj. Do ponto de vista da literatura dedicada às nossas ciências sociais, a busca por princípios teóricos unificadores ou origens sociais similares impediu a bibliografia de identificar uma forma compartilhada de fazer sociologia na primeira geração do instituto. Como tentamos mostrar, mais que referências biográficas ou bibliográficas comuns, essa geração materializou sua concepção de sociologia política por meio de um arranjo organizacional-curricular centrado na especialização. Contudo, tal especialização não veio descolada de uma agenda maior de atuação, focalizada em três eixos: transição de uma agenda desenvolvimentista para outra atenta às múltiplas dimensões das desigualdades que obstaculizavam o processo de democratização social brasileiro; ênfase em uma formação acadêmica plural sem, contudo, deslocá-la das questões candentes na época; e atuação política na condição de *experts* junto a agências estatais e, também, a movimentos da sociedade civil.

## Data Availability

https://50anos.iesp.uerj.br

## NOTAS

1 Vejam-se, em especial, os ensaios reunidos por Toledo (2005).

2 Parte desse acervo pode ser acessada digitalmente em: http://50anos.iesp.uerj.br.

3 Que esses colegas compartilhassem históricos de formação e agendas de pesquisa não significa, por certo, que estivessem livres de fortes tensões interpretativas; veja-se, por exemplo, a oposição entre Bolívar Lamounier e Wanderley Guilherme dos Santos, estampada em “As eleições e a dinâmica do processo político brasileiro” (Santos, 1977), travada ao redor da natureza dos votos de oposição em 1974.

4 Para as disputas ao redor de Florestan, vejam-se: D’Incao (1987); Ianni (2004); Romão (2006); Rodrigues (2005). Em outra chave, ele é divorciado de seus engajamentos fazendo “da Universidade o espaço único de sua autoconstrução” (Arruda, 2010, p.15).

5 Além de empiricamente limitada, essa abordagem torna-se teoricamente contraditória ao não submeter o próprio olhar do sociólogo às suas premissas teóricas. Nos termos de Boltanski e Thevenot (1991, p.24), essa sociologia crítica conjuga uma concepção altamente positivista da neutralidade do sociólogo com a exigência de que ele assuma uma missão altamente crítica perante os campos que estuda. Logo, se o “campo da sociologia” é visto como espaço de disputas incessantes por legitimidade e prestígio intelectual, o sociólogo que enuncia esses diagnósticos deve ser entendido como um cientista positivo e rigoroso.

6 Tal duplicidade é também afinada à sua sociologia da religião, pois “*Beruf =* dependendo da ênfase contextual, será traduzido ou por vocação ou por profissão; quando for o caso de dar destaque à imbricação dos dois registros, usaremos: vocação profissional, ou então: profissão como vocação” (Pierucci, 2005, p.18-19).

7 Com efeito, os investimentos da Fundação Ford não foram exclusivamente orientados para o Iuperj, visando a organizações também de outros estados, como Minas Gerais, São Paulo e, mesmo, Rio Grande do Sul. Sobre a Ford e a ciência política brasileira, ver Canêdo (2018), Rodrigues (2020) e Amorim (2021).

8 Em entrevista ao Centro de Pesquisa e Documentação de História Contemporânea do Brasil da Fundação Getulio Vargas, Sergio Miceli (2019, p.5) traduz em detalhe como esse sistema funcionava na prática: “A sala dele [Florestan Fernandes] tinha uma tábua, um quadro negro atrás, com os nomes dos objetos disponíveis para a cadeira de sociologia I. Ele disse: ‘Você pode ler e escolher um desses temas’. E eu disse: ‘Mas só esses?!’ Aí, lá no finalzinho, tinha assim: um negócio do Iseb. Aí eu disse: ‘Eu quero fazer então o Iseb’. Aí, eu escolhi o Iseb”.

9 O Iuperj foi alvo de repressão e infiltração constantes entre as décadas de 1960 e 1980. A lógica de perseguição ao Iuperj é, em boa medida, tributária do fechamento e da cassação do Iseb, cujo Inquérito Policial Militar, n.481, contém nada menos que trinta volumes. Para a visão contraditória pela qual o Iuperj era vigiado pelos aparelhos repressivos, remetemos a Czajka (2009) e Arquivo Nacional (1980).

10 Cabe notar que, ao final da década de 1980, Schwartzman (1988) desenvolveu dura crítica à produção brasilianista cuja reflexão espelhava e projetava no Brasil uma concepção de totalidade, àquela altura, a seu ver, superada nas ciências sociais.

11 Ver “Estado e sociedade: a obra de R. Boschi e E. Diniz” (Szwako, Moura, D’Avila, 2016). Para o par análogo “política e sociedade”, veja-se o mais recente curso *lato sensu* no âmbito do atual Iesp, bem como o nome da Coleção Iesp/Eduerj.

12 Ver “A rigor só existiu uma vida acadêmica na acepção das experiências europeias e norte-americana na Universidade de São Paulo, entendendo-se por isso uma atividade profissional permanente de docentes e pesquisadores em condições de fazer da universidade o centro de sua vida” (Miceli 2001, p.107). Em sua visão, “o Rio de Janeiro estava para a política assim como São Paulo estava para a ciência” (p.110). Arruda (1995, p.115) o segue de perto: “Foi a partir da fundação da Universidade de São Paulo que se pôde reproduzir o campo de ação dos cientistas sociais”; e, no ápice desse processo, estaria Florestan Fernandes – alçado a posição de um Sartre brasileiro (p.127) – cujas análises teriam tido o objetivo “de erigir o campo da disciplina sociológica” (p.145).
